# Trehalose Polyphleates, External Cell Wall Lipids in *Mycobacterium abscessus*, Are Associated with the Formation of Clumps with Cording Morphology, Which Have Been Associated with Virulence

**DOI:** 10.3389/fmicb.2017.01402

**Published:** 2017-07-25

**Authors:** Marta Llorens-Fons, Míriam Pérez-Trujillo, Esther Julián, Cecilia Brambilla, Fernando Alcaide, Thomas F. Byrd, Marina Luquin

**Affiliations:** ^1^Departament de Genètica i de Microbiologia, Facultat de Biociències, Universitat Autònoma de Barcelona Barcelona, Spain; ^2^Servei de Ressonància Magnètica Nuclear and Departament de Química, Universitat Autònoma de Barcelona Barcelona, Spain; ^3^Servei de Microbiologia, Hospital Universitari de Bellvitge-IDIBELL, Universitat de Barcelona Barcelona, Spain; ^4^The University of New Mexico School of Medicine, Albuquerque NM, United States

**Keywords:** *Mycobacterium abscessus*, trehalose polyphleates, CORDS, clumps, rough morphotypes, smooth morphotypes, virulence factors

## Abstract

*Mycobacterium abscessus* is a reemerging pathogen that causes pulmonary diseases similar to tuberculosis, which is caused by *Mycobacterium tuberculosis*. When grown in agar medium, *M. abscessus* strains generate rough (R) or smooth colonies (S). R morphotypes are more virulent than S morphotypes. In searching for the virulence factors responsible for this difference, R morphotypes have been found to form large aggregates (clumps) that, after being phagocytozed, result in macrophage death. Furthermore, the aggregates released to the extracellular space by damaged macrophages grow, forming unphagocytosable structures that resemble cords. In contrast, bacilli of the S morphotype, which do not form aggregates, do not damage macrophages after phagocytosis and do not form cords. Cording has also been related to the virulence of *M. tuberculosis*. In this species, the presence of mycolic acids and surface-exposed cell wall lipids has been correlated with the formation of cords. The objective of this work was to study the roles of the surface-exposed cell wall lipids and mycolic acids in the formation of cords in *M. abscessus*. A comparative study of the pattern and structure of mycolic acids was performed on R (cording) and S (non-cording) morphotypes derived from the same parent strains, and no differences were observed between morphotypes. Furthermore, cords formed by R morphotypes were disrupted with petroleum ether (PE), and the extracted lipids were analyzed by thin layer chromatography, nuclear magnetic resonance spectroscopy and mass spectrometry. Substantial amounts of trehalose polyphleates (TPP) were recovered as major lipids from PE extracts, and images obtained by transmission electron microscopy suggested that these lipids are localized to the external surfaces of cords and R bacilli. The structure of *M. abscessus* TPP was revealed to be similar to those previously described in *Mycobacterium smegmatis*. Although the exact role of TPP is unknown, our results demonstrated that TPP are not toxic by themselves and have a function in the formation of clumps and cords in *M. abscessus*, thus playing an important role in the pathogenesis of this species.

## Introduction

Nontuberculous mycobacteria are species of the *Mycobacterium* genus other than the *Mycobacterium tuberculosis* complex and *Mycobacterium leprae*. The overall prevalence of lung disease related to NTM is increasing worldwide and is caused by *Mycobacterium avium* complex, *Mycobacterium kansasii*, and increasingly, *Mycobacterium abscessus*, which is the species most commonly implicated in human pulmonary disease (McShane and Glassroth, 2015; Brown-Elliott and Philley, 2016; Koh et al., 2017). Similarly to *M. tuberculosis*, the agent of human tuberculosis, *M. abscessus* induces the production of granulomas and persists inside granulomas, developing caseous lesions in pulmonary tissue (Medjahed et al., 2010). *M. abscessus* pulmonary infections are of special importance in patients with underlying respiratory diseases such as bronchiectasis and cystic fibrosis (McShane and Glassroth, 2015; Brown-Elliott and Philley, 2016; Koh et al., 2017). In some of these patients, the therapeutic treatment is ineffective, and they experience chronic infections for long periods of time, frequently with a fatal outcome.

*Mycobacterium abscessus* strains isolated from humans form smooth (S) or rough (R) colonies when grown on agar medium. S colonies exhibit a bright and moist texture related to the presence of cell surface-exposed glycopeptidolipids (GPL), whereas R colonies are characterized by an irregular dry surface with many wrinkles and crests and are devoid of GPL (Howard et al., 2006; Nessar et al., 2011; Howard, 2013). Various studies have reported that R morphotypes produce the most severe illness in humans (Sanguinetti et al., 2001; Catherinot et al., 2007, 2009; Jönsson et al., 2007). In a recent and very interesting study, 50 serial isolates from nine patients with persistent *M. abscessus* infections have been analyzed on the basis of colony morphology (Park et al., 2015). The authors have found that R isolates predominate at later times during the course of the disease (median follow-up was 8 years). In six out of the nine patients, the colony morphology of the serial isolates was initially S before becoming predominantly R. Serial isolates from the other three patients showed R colony morphology throughout the course of the disease.

The increasing clinical importance of *M. abscessus* has piqued the interest of several groups of researchers who have developed different animal models with which to study the pathogenesis of this species (for a recent review, see Bernut et al., 2017). In these studies, R morphotypes have been found to be hyperlethal for mice and zebra fish embryos, whereas S morphotypes are unable to produce infection (Byrd and Lyons, 1999; Howard et al., 2006; Bernut et al., 2014; Roux et al., 2016). The observations from human and animals indicate that the ability to switch between S and R morphotypes allows *M. abscessus* to transition between a colonizing phenotype and a more virulent, invasive form.

It is therefore of great importance to determine what factors allow the morphotype R to be much more virulent than the S morphotype.

Both morphotypes are able to grow inside macrophages but exhibit distinct behaviors. As it is possible to see in the study performed by Brambilla et al. (2016), phagosomes of macrophages infected with R morphotype contain more than one bacillus at 3 h post-infection (h.p.i), and of those, up to 30% of phagosomes contain clumps of more than five bacilli. In contrast, phagosomes of macrophages infected with the S morphotype contain mainly isolated bacilli (Byrd and Lyons, 1999; Brambilla et al., 2016; Roux et al., 2016). This distinct fate has been related to the highly aggregative nature of R morphotype bacilli, which grow close together, leave no spaces among them and form large aggregates (clumps) (Sánchez-Chardi et al., 2011; Brambilla et al., 2016). At 48–72 h.p.i, macrophages infected with the R morphotype are destroyed, thus releasing large clumps of bacilli outside the cells; however, macrophages infected with the S morphotype are unaffected. Using zebra fish embryos it is possible to visualize as extracellular clumps, which grow extensively forming cords (cording), that were unphagocytable for macrophages and neutrophils promoting rapid larval death (Bernut et al., 2016). In this model, the S morphotype is unable to produce infection. In addition, an R mutant defective in cording exhibits impaired replication in zebra fish (Halloum et al., 2016). These results relate clumps of cording morphology to virulence in *M. abscessus*, and, interestingly, cording has also been related to virulence in *M. tuberculosis*, a species with a very stable R morphotype (Middlebrook et al., 1947).

Cords are snake-like structures that are formed through end-to-end and side-to-side aggregation of bacilli, in which the orientation of the long axis of each cell is parallel to the long axis of the cord (Julián et al., 2010). Microscopic cords were described for the first time by Robert Koch in *M. tuberculosis* (Koch, 1882). Various studies performed with natural and constructed mutants of *M. tuberculosis* have confirmed the correlation between cording and virulence in this species (for a review, see Glickman, 2008). Some of *M. tuberculosis* constructed mutants defective in cording exhibit altered structures of their mycolic acids (Glickman et al., 2000). Other *M. tuberculosis* non-cording mutants are defective in the production of some extractable glycolipids, but preserve mycolic acid structures and profiles (Glickman, 2008). In *M. abscessus*, cording was described for the first time in the 390R strain as well as the lack of cording of the related 390 S morphotype (Howard et al., 2006). An R mutant of *M. abscessus* defective in cording shows an altered ratio of α-mycolic acid to α’-mycolic acid but no differences in their structures (Halloum et al., 2016). Thus, to date there is not a unique candidate for cording. The cell wall of mycobacteria is very rich in complex lipids that interact with one another and with polysaccharides and proteins, thereby forming a definite and stable wall architecture (Daffé et al., 2014; Jankute et al., 2015), so it is logical to assume that the elimination or modification of one of these lipids may disrupt the original configuration by eliminating the organization in cords even if these lipids are not directly responsible for cording.

Another approach to shed new light on the cell wall components responsible for cording, following the pioneering studies performed by Bloch, is to disrupt cords with organic solvents and analyze the lipids present in the organic extracts (Bloch, 1950).

The objective of this work was to identify the compounds responsible for cording in *M. abscessus* by breaking the cords with PE and identifying the lipids present in the PE extracts. Furthermore, because mycolic acids are a candidate responsible for cording, we studied the composition and structure of mycolic acids of R (cording) and S (non-cording) morphotypes that were derived from the same parent strain. The localization of candidate lipids on the surfaces of cords and bacilli was accomplished by TEM.

## Materials and Methods

### Mycobacterial Strains and Growth Conditions

The bacterial strains used in this study were *M. abscessus* 390, *M. abscessus* type strain DSMZ 44196^T^, and a clinical isolate of *M. abscessus* BE48. S and R morphotypes of *M. abscessus* 390 and DSMZ 44196^T^ were obtained in previous studies (Byrd and Lyons, 1999; Brambilla et al., 2016). For the clinical isolate *M. abscessus* BE48, the strain was initially R, but we obtained the S morphotype after several passages on agar medium. All strains were grown in Middlebrook 7H9 broth (Difco, United States) for pellicle formation and in Middlebrook 7H9 agar (Difco, United States) for colony morphology observation and mycolic acid extraction. One liter bottles with 100 ml of Middlebrook 7H9 broth were inoculated with 1 ml of bacterial suspension, adjusted to No. 1 of the McFarland standards of turbidity. Pellicles of *M. abscessus* grew at the air-medium interface of Middlebrook 7H9 broth at 37°C.

### Mycolic Acid Extraction and Purification

For extraction and methylation of mycolic acids, bacteria scraped from Middlebrook 7H9 agar plates were subjected to an acid methanolysis procedure (Minnikin et al., 1980). Briefly, 50 mg of scraped bacteria were treated with 2 ml of methanol, toluene and sulfuric acid (30:15:1; v:v:v) and heated at 80°C overnight, and the samples were then extracted twice with *n*-hexane. The *n*-hexane extracts that contained the methyl mycolates were evaporated to dryness at 40°C under nitrogen stream. The mycolates were concentrated by precipitation in cold methanol (4°C, overnight) and analyzed by conventional TLC on silica gel-coated plates (G-60, Merck, Germany). The 10 μl of each sample was loaded to the TLC and plates were developed with *n*-hexane/diethyl ether (85:15; v:v, three runs). The mycolates were observed as dark spots after the plates were sprayed with phosphomolybdic acid (VWR, United States; 10% in ethanol) and charred at 120°C. Purification of mycolates was performed by using preparative TLC plates with a concentration zone (G-60, Merck, Germany) that were developed as described above. Mycolates were visualized with iodine vapors, scraped from TLC plates and recovered with diethyl ether. The purification steps were monitored by TLC as described above.

### Analysis of Mycolic Acids by Nuclear Magnetic Resonance Spectroscopy

Purified dried mycolates were dissolved in 600 μl of CDCl_3_ (99.80% D, Cortecnet, France) and transferred to 5-mm-diameter NMR tubes. NMR experiments were recorded on a Bruker Avance II 600 (Bruker Biospin, United States) equipped with a 5 mm TBI probe with Z-gradients that operated at a ^1^H NMR frequency of 600.13 MHz and at 298.0 K. 1D ^1^H NMR spectra were acquired using a standard 90° pulse sequence with an acquisition time of 1.71 s and a relaxation delay of 3 s. Data sets were collected as 32k data points with a spectral width of 9590 Hz and as the sum of 128 transients. The resulting free induction decays were Fourier transformed, manually phased, and baseline corrected. All spectra were calibrated using the residual solvent signal (CHCl_3_) at a chemical shift (δ) of 7.27 ppm. The relative molar ratios of characteristic molecular moieties were determined by the integration of representative resonances.

### Scanning Electron Microscopy

The spreading pellicles that formed on the surface of the liquid medium were collected with a 0.2 μm nuclepore membrane (Whatman, United Kingdom) and processed for analysis by scanning electron microscopy (SEM) as previously described (Brambilla et al., 2016). Briefly, pellicles were fixed in 2.5% (vol/vol) glutaraldehyde in 0.1 M phosphate buffer (pH 7.4) for 2 h at 4°C and then washed four times for 10 min each in 0.1 M phosphate buffer. Then, the samples were post-fixed in 1% (wt/vol) OsO_4_ and 0.7% ferrocyanide in phosphate buffer and washed with water. This process was followed by dehydrating the samples in an ascending ethanol series (50, 70, 80, 90, and 95% for 10 min each and twice with 100% ethanol) and subsequently critical-point drying the samples with CO_2_. Finally, the samples were coated with gold and observed using an SEM EVO (Zeiss, Germany) at 15 kV.

### Observation of Cords and Clumps after Application of PE to *M. abscessus* Pellicles

After 2 weeks of growing in Middlebrook 7H9 broth, *M. abscessus* formed a consistent pellicle at the air-medium interface. A superficial lipid extract of these pellicles was performed by using a modification of Bloch’s protocol (Bloch, 1950). Briefly, pellicles of *M. abscessus* were filtered. Then, avoiding the complete drying of the pellicles, we placed 1.5 g of each sample in a beaker with 40 ml of PE (40–60°C b.p.) and agitated them for 5 min. After that, the PE extracts were filtered and evaporated. Then, one part of the cellular residue formed by the treated bacteria was recovered in a tube with glass beads, which was shaken for 10 min. Then, 2 ml of PBS was added, the tube was shaken for 10 s, and three drops of the suspension were placed in a slide and left to dry. As a control, bacteria were treated with the same protocol but without the PE extraction step. All the samples were stained using Ziehl-Neelsen stain. Another part of the cellular residue was plated in agar plates to test the viability of the treated bacteria by CFUs count and to observe the colonial morphology. After growing, these treated bacteria were inoculated in Middlebrook 7H9 broth and the biopellicles formed were extracted with PE as described above.

### Analysis of the Lipidic Components of the PE Extract

A series of TLCs were performed to study the lipidic profile of the PE extracts, using 10 μl of all samples for each TLC. For unidimensional TLCs, the mobile phases that were used were PE 60–80°C/diethyl ether (90:10, v/v), chloroform/methanol (85:15, v/v), and chloroform/methanol/water (60:35:8, v/v/v; and 90:10:1, v/v/v). For bidimensional TLCs, the mobile phase used were first direction PE 60–80°C/ethyl acetate (98:2, v/v, thrice), second direction PE 60-80°C/acetone (98:2, v/v, once); first direction PE 60-80°C/acetone (98:2, v/v, thrice), second direction toluene/acetone (95:5, v/v, once); first direction chloroform/methanol (96:4, v/v, once), second direction toluene/acetone (80:20, v/v, once); and first direction chloroform/methanol/water (100:14:0.8, v/v/v, once), second direction chloroform/acetone/methanol/water (50:60:2.5:3, v/v/v/v, once). Compounds in the TLC plates were revealed by using anthrone (Merck, Germany; 1% in sulfuric acid) or phosphomolybdic acid (VWR, United States; 10% in ethanol) and then heating the plate at 120°C or by using molybdenum blue (Sigma, United States) without heat.

### Purification and Structural Characterization of Compounds X, Y, Z and Triacylglycerides by NMR and Mass Spectrometry

Compounds X, Y, Z and TAGs were purified by column chromatography. Approximately 50 mg of PE extract obtained as described above was added to a Silica Gel 60 (Merck, Germany) column. A series of solvent mixtures of PE 60–80°C with increasing concentrations of diethyl ether was used for the elution of the PE-extracted components.

Purified compounds were dissolved in 600 μL of CDCl_3_ (99.80% D, Cortecnet, France) and analyzed by NMR spectroscopy. The equipment described operating at ^1^H and ^13^C NMR frequencies of 600.13 and 150.90 MHz, respectively, and at 298.0 K, was used. 1D ^1^H spectra of the three compounds were acquired and processed using the same parameters previously described. 2D ^1^H,^1^H-COSY; ^1^H,^13^C-HSQC; ^1^H,^13^C-HMBC; and ^1^H-DOSY experiments were performed using standard Bruker pulse sequences and acquired under routine conditions. Chemical shifts were referenced to the residual solvent signals (δ_H,_ 7.26 and δ_C,_ 77.0 ppm). Proton signal multiplicity is indicated in the text by d (doublet), t (triplet), dd (double doublet) and m (multiplet). In the case of compounds Y and Z, standard 1D ^31^P spectra were also recorded.

For MS, purified compounds X, Y, and Z were dissolved in 50 μl of chloroform/methanol (2:1, v/v). This suspension was mixed with a matrix made of 10 mg/ml 1,8,9-anthracenetriol (dithranol) at a 1:1 ratio, and 1 μl of the mix was deposited on a ground steel plate. The sample was analyzed using a negative polarity reflectron and an acceleration voltage of 25 kV in a MALDI-TOF UltrafleXtreme (Bruker Daltonics, United States). The calibration was performed using external calibrators (Bruker Daltonics, United States).

### Study of the *M. abscessus* Cell Wall by TEM

*Mycobacterium abscessus* pellicles were collected in sterilized filters, passed to a tube and processed for TEM observation. Two processes were used to observe the samples: the conventional preparation, as described by Bleck et al. (2010); and the OTO method, as described by Seligman et al. (1966). Briefly, samples for the conventional preparation were fixed in 4% FA and 5% GA in 0.1 M HEPES (4-(2-hydroxyethyl)piperazine-1-ethanesulfonic acid) buffer (pH 7.4). Post-fixation was performed with 2% OsO_4_ in PBS for 1 h at room temperature. Then, the samples were dehydrated in an ascending ethanol series and post-stained with saturated uranyl acetate at the 90% ethanol step for 30 min at 37°C. Bacteria were finally embedded in epoxy resin, and ultrathin sections were made (Bleck et al., 2010). For the OTO method, ultrathin sections that were previously fixed with OsO_4_ but were not post-stained with uranyl acetate were exposed to a 1% hot aqueous solution of TCH (thiocarbohydrazide) for 1 h at 50°C, and this was followed by four to eight washes with hot water. Then, the sections were again exposed to OsO_4_ (Seligman et al., 1966; Hall et al., 2012). This method results in further deposition of osmium on the samples and increases lipid contrast (Belazi et al., 2009). All samples were observed using a JEOL 1400 (Japan) TEM equipped with a Gatan ES1000W Erlangshen CCD (United States) camera.

### Coating of Beads and the Study of Bead Organization after Coating

Amine-modified yellow–green latex beads (Sigma, United States) with an average diameter of 1 μm were used and coated with TPP A by using variations of the protocols described by Kang and Schlesinger (1998) and Vergne et al. (2004). Briefly, 5 μl of bead suspension at an initial concentration of 4.6 × 10^10^ beads/ml were transferred to a tube and these beads washed twice with 0.05 M carbonate-bicarbonate buffer (pH 9.6) and coated with purified TPP obtained from the R morphotype of the *M. abscessus* 390 strain and maintained in DMSO (Merck, Germany). Beads were incubated in 8% DMSO in PBS with 0.8 mg/ml of TPP for 2 h at 37°C with agitation. Control uncoated beads were treated with 8% DMSO in PBS without TPP. After the 2 h incubation, the beads were washed twice with PBS and blocked with 5% BSA (Roche, Germany) in PBS for 1 h at 37°C with agitation to prevent non-specific binding. After that, beads were washed once with 0.5% BSA in PBS.

The successful coating with TPP was demonstrated by staining the beads with Nile Red (Sigma-Aldrich, United States). Briefly, 1 μl of 0,5 mg/ml Nile Red in ethanol was added to 50 μl of beads, which were incubated for 10 min at 37°C. Then, the beads were washed three times with PBS (Christensen et al., 1999), and 10 μl of the bead suspension was spread on a slide by using a coverslip. Nile Red-stained TPP-coated beads were observed with a CLSM using a TCS-SP5 CLSM (Leica, Germany) with a PlanApo 63 (numerical aperture [NA] 1.4) oil objective.

To determine the organization of TPP-coated and uncoated beads, images of the bead suspension were obtained using a CLSM and analyzed with ImageJ software (National Institutes of Health, United States). The analysis described three types of aggregates, depending on their area: fewer than 3 μm^2^, between 3 and 6 μm^2^, and more than 6 μm^2^ (Brambilla et al., 2016). The area covered by each type of aggregate was added, and the statistical analysis was performed to determine the percentages of area that were covered by each type of aggregate. All experiments were conducted three times.

### Study of Macrophage Viability When in Contact with Beads

J774 murine macrophage cell line was used for the experiments and maintained at 37°C in a humidified atmosphere of 5% CO_2_ in Dulbecco’s modified Eagle’s medium with L-glutamine and high glucose (DMEM) (Gibco, Austria). This media was supplemented with 10% heat-inactivated fetal bovine serum (FBS) (HyClone, United Kingdom), 100 U/ml penicillin G (LERN, Spain), and 100 μg/ml streptomycin (Reig Jofre, Spain), which was considered to be CM.

J774 macrophages cells were seeded into 96-well tissue culture plates (Thermo Fisher Scientific, Denmark) at a density of 1.5 × 10^4^ cells per well in CM and incubated for 24 h. Then, TPP-coated and uncoated beads were added on the macrophage cultures at an optimized ratio of 30 beads per macrophage (Brambilla, 2015). The medium was removed 3 h later, and the cultures were washed three times with PBS and then incubated with CM. At different time points (24, 48, and 72 h), culture supernatants were recovered, and the MTT assay was performed with the cells as described previously (Noguera-Ortega et al., 2016). Viability was calculated using the control wells that contained untreated cells as a reference representing 100% growth.

As a positive control for virulence, J774 macrophages were infected with *M. abscessus* 390R strain as previously described (Brambilla et al., 2016). To prove if PE-treatment had any effect in the virulence of the bacteria, J774 macrophages were also infected with *M. abscessus* 390R PE-treated.

### Analysis of the Cytokine Production by Macrophages Treated with Beads

Supernatants of macrophages treated with beads were recovered at different time points, in the manner explained above, and centrifuged to eliminate macrophages that had detached from the well; the concentrations of TNF-α (R&D Systems, United States) and IL-6 (BD Biosciences, United States) were determined, following the manufacturer instructions.

### Study of the Phagolysosome Fusion in J774 Macrophages

J774 macrophages were seeded onto CLSM culture dishes (Mat Tech, United States) at a concentration of 2.5 × 10^5^ cells per dish. Those macrophages were cultured in DMEM with LysoTracker Red DND-99 (Life Technologies, United States) at a concentration of 1:10000. The 24 h after the seeding, cells were placed in contact with beads in order to compare the phagolysosome fusion between macrophages with uncoated beads versus macrophages with TPP-coated beads. After 24 h, dishes were observed using a TCS-SP5 CLSM (Leica, Germany) with a PlanApo 63 (NA, 1.4) oil objective, operating at a zoom of 2.5. One hundred macrophages were counted for each sample, and colocalization between LysoTracker and the green fluorescence produced by the beads was calculated using Pearson’s correlation coefficient.

### Statistical Analysis

Analysis of the percentage of aggregates and comparison of the macrophage viability with TPP-coated and uncoated beads were made using multiple *t*-tests. Statistical significance was determined using the Holm–Sidak method, an extension of the Holm–Bonferroni method, in Prism 6 (Version 6.01, GraphPad Software, United States). Differences were considered to be significant at *p* < 0.05.

## Results

### Both Morphotypes Had the Same Proportion of Mycolic Acids with no Differences in Structure

**FIGURE 1:**
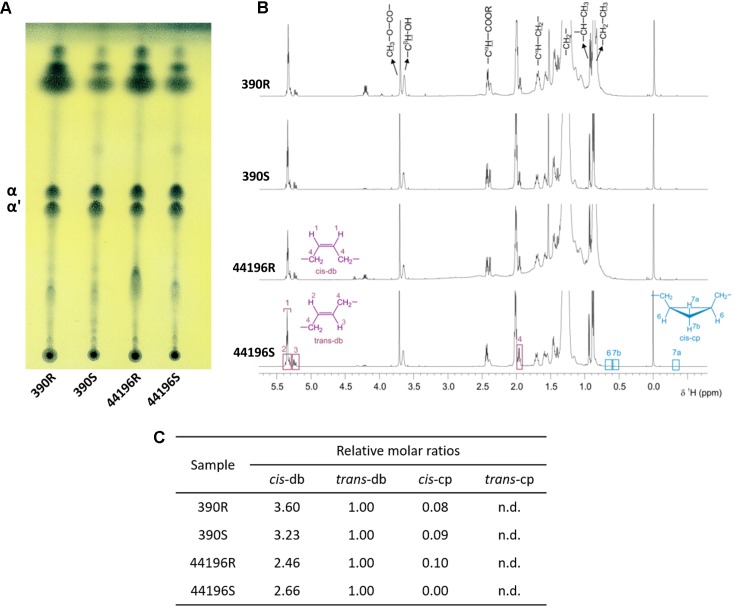
Structure of mycolic acids. **(A)** TLC analysis of mycolates from *M. abscessus* strains. TLC was developed with *n*-hexane-diethyl ether at 85:15 (three runs). α (α-mycolates) α’ (α’-mycolates). **(B)** ^1^H NMR spectra of purified mycolates from *M. abscessus* strains. Spectra acquired at a temperature of 298.0 K and in a magnetic field of 600.13 MHz. **(C)** Relative molar ratios of molecular moieties *cis*-db, *trans*-db, *cis*-cp, and *trans*-cp of mycolates from *M. abscessus;* db (double bonds), cp (cyclopropane ring), n.d. (not detected).

When the mycolic acid profiles of S and R morphotypes of *M. abscessus* 390 and 44196 were determined by TLC, no differences were detected in the ratios of α-mycolic and α’-mycolic acids. In both strains, the two morphotypes produced similar amounts and ratios of the mycolic acids (**Figure 1A**). As shown in **Figure 1B**, the NMR analysis indicated that both strains had a very similar spectrum for purified mycolic acid methyl esters. In a comparison of the relative molar ratios, samples from the R and S morphotypes showed the same average of *cis* and *trans* double bonds, and cyclopropane rings were detected only in trace amounts (**Figure 1C**).

### R Morphotypes Produced Rough Pellicles in Which Bacilli Organized into Cords

**FIGURE 2:**
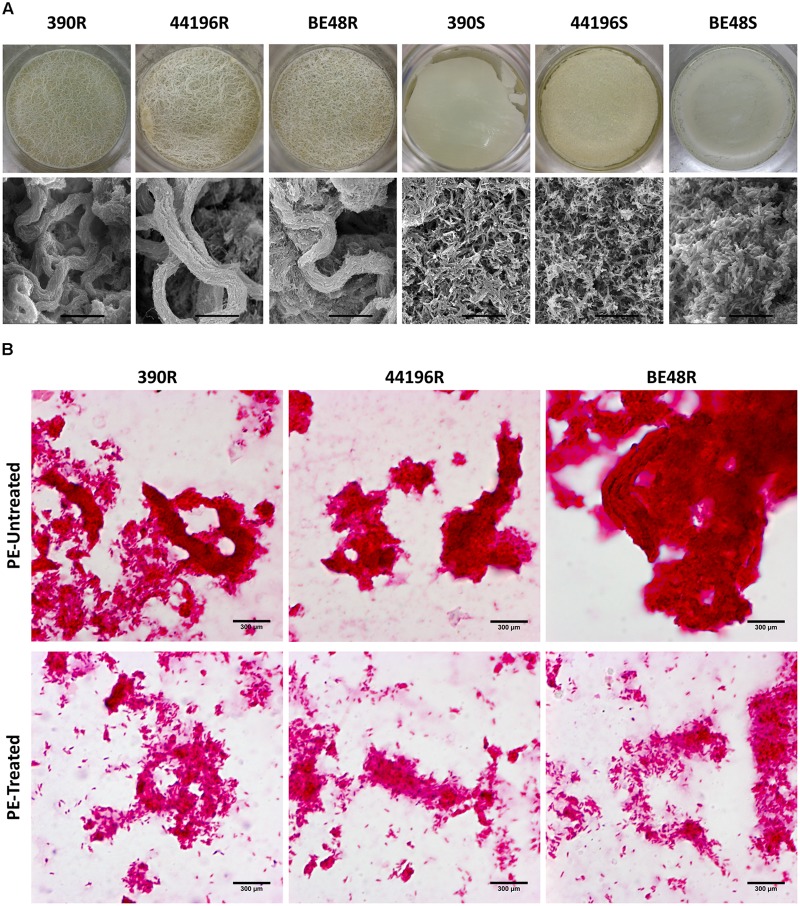
Pellicles and microscopic cords of untreated bacteria, and cord disorganization in PE-treated bacteria. **(A)** Images of the pellicles formed by all morphotypes of *M. abscessus* in 1 L bottles with 100 ml of medium, and their ultrastructure, as determined by SEM. Bar size 15 μm. **(B)** Observation by optical microscopy of untreated and treated with PE for 5 min samples, both stained using the Ziehl-Neelsen method. Bar size 300 μm.

All R morphotypes (390R, 44196R, and BE48R), when grown on liquid media, produced thick and wrinkled pellicles with structures that resembled macroscopic cords. When observed with SEM, pellicles from the R morphotype showed an organization of their bacilli that was typical of that in cord-forming mycobacteria (**Figure 2A**). In contrast, all S morphotypes (390S, 44196S, and BE48S) produced a thin pellicle with a flat surface. No organization of the bacilli in clumps or cords was observed when the ultrastructure of these pellicles was observed by SEM (**Figure 2A**).

### Cord Disorganization Was Observed after Treatment of Pellicles of R Morphotypes with PE

Pellicles of R morphotypes that were untreated or treated with PE were observed by using optical microscopy after they were stained with the Ziehl-Neelsen method. Bacteria in the untreated samples were aggregated, forming clumps and cords, and only a few bacteria were solitary (**Figure 2B**). However, disintegration of clumps and cords was observed in samples treated with PE. In these samples, higher amounts of free bacteria in all the fields monitored from each preparation were clearly observed. Although in some points there were still some aggregates, the disorganization of the clumps and cords was evident (**Figure 2B**).

Regarding viability, no effect of the PE treatment was observed in the CFU count. The viability of the bacteria was not affected for the extraction, and the same occurred with the colonial morphology.

### TPP Were Detected in the PE Extract from the R Morphotypes

**FIGURE 3:**
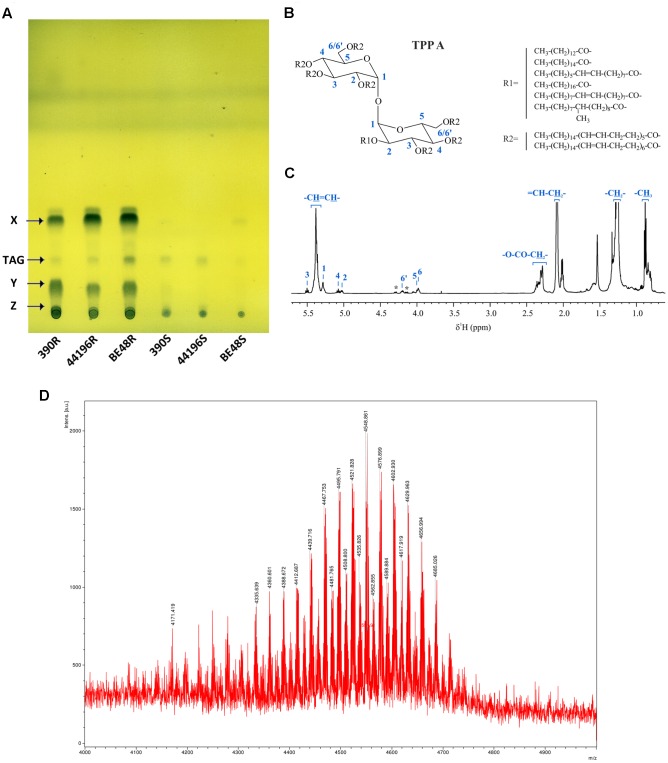
Detection and characterization of compound X. **(A)** TLC of all PE extracts. The solvent system used was PE 60-80°C/diethyl ether (90:10, v/v), and the plates were revealed with 10% phosphomolybdic acid. X (Compound X), TAG (Triacylglycerides), Y (Compound Y), Z (Compound Z). **(B)** Chemical structure of Compound X purified from *M. abscessus* 390R. Length and structure of R1 and R2 were obtained from Burbaud et al. (2016). **(C)** ^1^H NMR spectra of compound X in CDCl_3_ with peaks corresponding to the TPP A assignment (asterisks denote signals of TAG impurities). **(D)** MALDI-TOF mass spectrum of compound X (region between *m/z* 4000 and 5000 is magnified).

Organic material was detected only when PE extracts from R morphotypes were developed in unidimensional TLC eluted with PE 60–80°C/diethyl ether (90:10, v/v) and bidimensional TLC eluted with PE 60–80°C/ethyl acetate (98:2, v/v, thrice, first direction) and PE 60–80°C/acetone (98:2, v/v, once, second direction). When PE extracts were monitored with other TLC developing systems, no organic material was observed. In samples from R morphotypes, two relevant compounds (X and Y) were observed when unidimensional TLC plates were revealed with phosphomolybdic acid (**Figure 3A**). After PE extracts were fractionated on a silica gel column, a third compound that was retained at the point of sample application in TLC was purified (compound Z) (Supplementary Figure S1). Only trace amounts of compound X were detected in samples from 390S and BE48S strains (**Figure 3A**), and none of the compounds were detected in the 44196S extract. Trace amounts of TAG were detected in all the morphotypes. TAG of the 390R strain were purified and identified by NMR (Supplementary Figure S2). No other lipidic compounds were detected in these PE extracts. When extraction with PE was extended from 5 to 15 min, compound X spots showed more intensity in S morphotype samples, thus suggesting that S morphotypes can synthesize this compound but that it is not exposed on the surface (Supplementary Figure S3). No differences in the lipidic profile were observed in the PE extracts from bacteria cultured after being extracted.

Purified compound X was identified by NMR spectroscopy and MS as TPP type A (**Figure 3B**). The identification was made on the basis of comparison with previous data (Burbaud et al., 2016). **Figure 3C** shows the ^1^H NMR spectrum of TPP A. The characteristic ^1^H and ^13^C resonances of TPP A glucosyl units were clearly observed. A doublet at 5.28 ppm corresponding to anomeric proton H1, the characteristic triplet at 5.50 ppm corresponding to H3 and peaks at 5.07 (t), 5.03 (dd), 4.20 (m), 3.98 (m) and 3.97 (m) ppm corresponding to H4, H2, H6, H6’, and H5, respectively, were identified. ^1^H,^1^H-COSY correlations, as well as carbon resonances of glucosyl units, obtained from ^1^H,^13^C-HSQC and ^1^H,^13^C-HMBC spectra, were in accordance with the TPP A structure and with previously described values. The intense signals corresponding to the polyunsaturated fatty acyl substituents are indicated in the figure. The MALDI-TOF MS analysis further confirmed the identity of the molecule (**Figure 3D**) (Burbaud et al., 2016). Residual TAG was detected by both NMR and MS (indicated with asterisks in the ^1^H spectrum on **Figure 3D**) (More detail in Supplementary Figure S4).

Compounds Y and Z were isolated and analyzed separately by NMR spectroscopy and MS. The concerted analysis of the NMR spectra allowed for their ^1^H and ^13^C NMR characterization (Supplementary Figures S5, S6). Both compounds were identified as TPP molecules. The ^1^H spectra and 2D correlations showed the same signals as TPP A plus some new peaks. In both cases, the presence of a characteristic multiunsaturated system (broad peak at δ_H_ 5.4 ppm correlated via HSQC to a peak at δ_C_ 127–130 ppm), as well as methylene (δ_H_ 1.2–1.3 ppm) and methyl groups (δ_H_ 0.9 ppm) characteristic of alkyl chains were observed. The same ^1^H and ^13^C NMR signals corresponding to the TPP A trehalose unit were exhibited in spectra of Y and Z, thus suggesting that they have a common glucosyl residue. In the case of compound Y, H3 (3.54 ppm) and H4 (3.82 ppm) of the second glucosyl residue were strongly shielded compared with those of TPP A (5.50 and 5.07 ppm, respectively), which suggests that position 3 and/or 4 are not acylated. Similarly, H4 (3.82 ppm), H6 (3.58 ppm) and H6’ (3.53 ppm) of the second glucosyl residue of Z were strongly shielded compared with analogous protons of TPP A (5.07, 4.20, and 3.98 ppm, respectively), which suggests that position 4 and/or 6 are not acylated. These results were supported by MS analyses (Supplementary Figure S7). Compounds Y and Z yielded analogous MALDI-TOF MS spectra, which indicates that they are structural isomers. Their mass also suggested the lack of one R2 substituent compared with TPP A (an envelope of peaks between m/z 4093 and 3849 for Y and Z, in contrast with m/z 4596 and 4297 for TPP A).

### R Morphotypes Had an Electrodense Material in the Outer Layer of the Cell Wall

**FIGURE 4:**
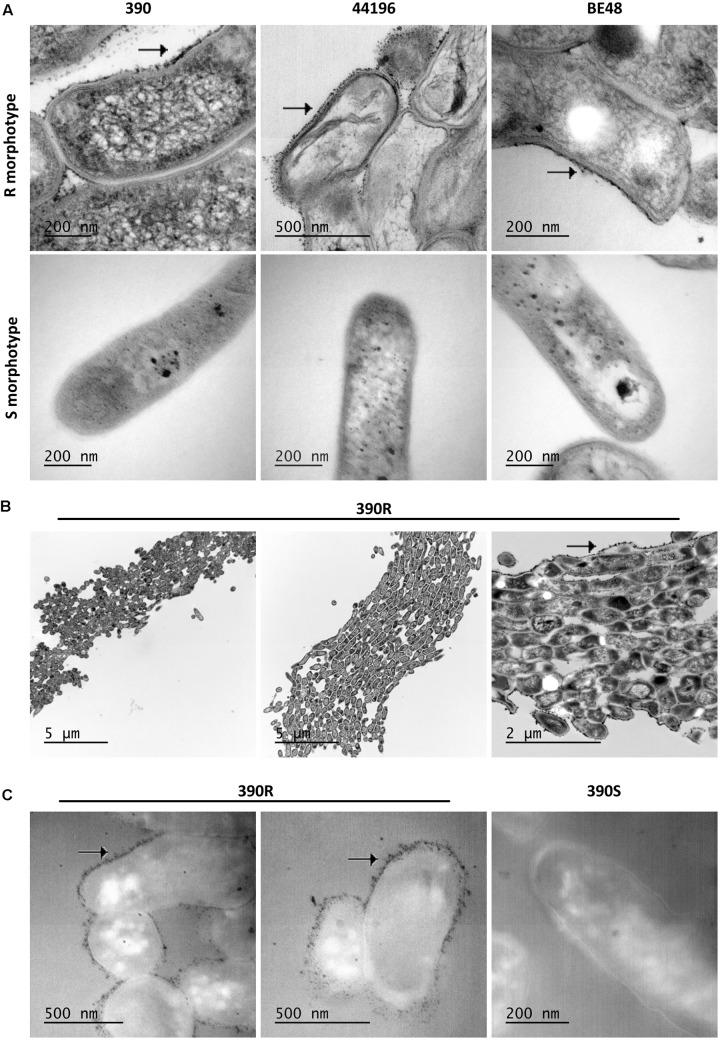
An electrodense material is observed on the cell wall of R morphotypes. **(A)** Images obtained by TEM of *M. abscessus* bacilli. Arrows indicate the accumulation of electrodense material on the wall of the R morphotype. **(B)** Example of cord formation in *M. abscessus* 390R. Arrows indicate the accumulation of electrodense material outside the cord. **(C)** Images of OTO staining, showing an important deposit of OsO_4_ in the R morphotype and no deposits in the S morphotype. Images from *M. abscessus* 390R. Similar images were obtained from the other strains.

When bacilli of the R and S morphotypes of *M. abscessus* were visualized by TEM, some electrodense layers and some electrotransparent layers were detected on their cell wall. It was of special interest that electrodense material irregularly accumulated in the outer layer of the bacilli from the R morphotype (**Figure 4A**), whereas this material did not accumulate in the S morphotype cell wall (**Figure 4A**). When a minor magnification of the TEM images was analyzed, the cord organization in the R morphotype and the accumulation of this electrodense material outside the cord were observed (**Figure 4B**).

The OTO stain provides a major contrast of the lipidic components because it augments the OsO_4_ deposit on these lipids. An important accumulation of OsO_4_ was observed outside the bacilli in R morphotypes from the samples treated with this stain, and no accumulation was observed in S morphotypes (**Figure 4C**).

### TPP-Coated Beads Formed Aggregates

**FIGURE 5:**
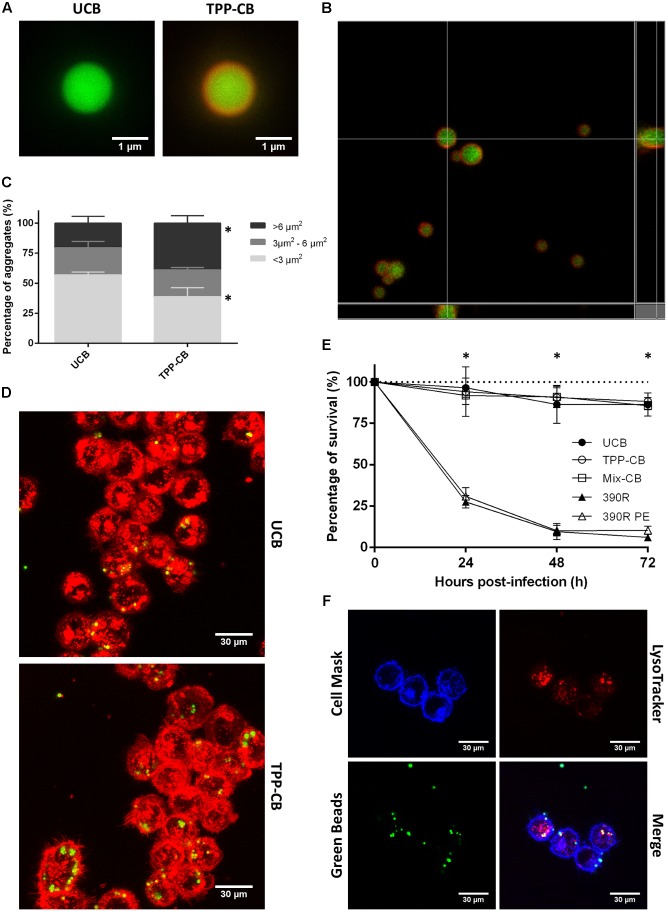
Beads are covered by TPP, and they do not have a toxic effect when in contact with macrophages. **(A,B)** Beads are in green and TPP-coating is observed as the red covering, owing to staining with Nile Red. Bar size in **(A)** 1 μm. **(C)** Results of the study of bead aggregation. ^∗^*p* < 0.05 in multiple *t*-test. The results represent the mean ± SD of triplicate preparations. **(D)** Example of the images obtained by CLSM that were used to study the aggregation of the beads; macrophages phagocytose both types of beads. Bar size 30 μm. **(E)** Viability of macrophages treated with uncoated beads; TPP A-coated beads; beads coated with TPP A, compound Y and Z; 390R *M. abscessus* strain and 390R *M. abscessus* PE treated. Significant differences were detected only between macrophages treated with beads and macrophages infected with bacteria (^∗^*p* < 0.05). The data are representative of one out of three independent experiments. **(F)** Images of colocalization of beads and acidic vesicles. Macrophages in blue stained with Cell Mask Deep Red, beads in green and acidic vesicles in red with LysoTracker Red. Bar size 30 μm. UCB (Uncoated beads), TPP-CB (TPP A-coated beads), Mix-CB (beads coated with TPP A, compound Y and Z), 390R (*M. abscessus* 390R stain), 390R PE (*M. abscessus* 390R treated with PE).

The coating of beads by TPP A was confirmed by CLSM, because Nile Red stained the lipids coating the beads (**Figures 5A,B**), and these beads appeared to be surrounded by red fluorescence. When observed by CLSM, the TPP A-coated beads presented more aggregation than uncoated beads. Statistical analysis showed significant differences between the areas of the aggregates of the two samples, thus indicating that TPP A-coated beads produced larger aggregates than uncoated beads (**Figure 5C**). The percentage of aggregates that were larger than 6 μm^2^ was 20.4% ± 5.7 (mean ± SD) for the uncoated beads and 38.9% ± 6.2 (*p* < 0.05) for the TPP A-coated beads. The difference in area between aggregates of uncoated beads that were smaller than 3 μm^2^ (57.0% ± 2.3 of the area covered) and aggregates of TPP A-coated beads of the same size (38.8% ± 7.5 of the area covered) was also significant (**Figure 5C**). These results are obtained from beads coated with TPP A. However, beads were coated also with a mix of the three compounds described by NMR (TPP A, Y, and Z), all TPP molecules, and no differences were observed between the results obtained with the beads coated with TPP A and those obtained with the beads coated with the mix of TPP molecules (TPP A, Y, and Z compounds) (data not shown).

### TPP-Coated Beads Had no Effect on the Viability of Macrophages

As observed with CLSM, macrophages did not distinguish between uncoated and TPP A-coated beads in phagocytosis (**Figure 5D**). Both types of beads were found inside the cells and in a similar ratio. J774 macrophages with phagocytized uncoated beads represented a percentage of 32.39% ± 5.08 from the total of counted macrophages, and macrophages with phagocytized TPP A-coated beads represented the 37.69% ± 9.73. Moreover, macrophage viability was not altered after the interaction with TPP A-coated beads. No significant differences were observed in the viability of macrophages in contact with uncoated beads or TPP A-coated beads (**Figure 5E**). When cytokine production was analyzed, no production of TNF-α and IL-6 was detected in macrophages treated either with uncoated or TPP A-coated beads (data not shown). Also related with the possible effect of TPP A on the viability of macrophages, no differences were observed when analyzing the colocalization of the beads inside the macrophages with acidic vesicles. Pearson’s correlation coefficient was of 0.40 ± 0.16 for uncoated beads and of 0.54 ± 0.15 for TPP A-coated beads (the results represent the mean ± SD of triplicate preparations) (**Figure 5F**). Similar results were obtained when macrophages were in contact with beads coated with the mix of TPP molecules (TPP A, Y, and Z compounds), that is no effect on the viability of the macrophages was observed (**Figure 5E**) and no production of TNF-α and IL-6 was detected (data not shown). Bacteria from R morphotype had the expected action in front of macrophages, as it has been already published (Brambilla et al., 2016). 390R strain killed all macrophages within 72 h. No difference between these results and the results obtained from bacteria PE-treated were observed (**Figure 5E**).

## Discussion

Cords are the first virulence factor described in *M. tuberculosis*. They were first observed by Robert Koch in 1882, and their significance increased in 1947 when studies by Middlebrook linked this phenotypic characteristic to the virulence of *M. tuberculosis* complex microorganisms (Middlebrook et al., 1947). In 1950, Hubert Bloch disrupted the cords in few minutes by using paraffin oil, PE, pure hexane and heptane but not with aqueous solutions. From these results, Bloch hypothesized that the substance responsible for the formation of cords might be a lipid and could be isolated from the extracts that were obtained with the abovementioned organic solvents (Bloch, 1950). Because cording was related to virulence, Bloch’s next objective was to identify a toxic substance in these extracts. Thus, from PE extracts, he purified a toxic compound that he named “cord factor” (Bloch et al., 1953). This toxic compound was found in very small proportions, representing approximately 1% of the total PE extract (Bloch et al., 1953). In collaboration with Noll, Bloch identified the toxic compound as trehalose-6,6′-dimycolate (TDM) (Noll et al., 1956). Hence, TDM was associated with the term “cord factor,” an error that has persisted and causes many mycobacteriologists to associate the formation of cords with the TDM compound. As Bloch himself explained, TDM was termed “cord factor” because it was obtained from “cord-forming” strains of mycobacteria and not because it was the compound responsible for the formation of such cords (Bloch et al., 1953). Agreeing with Bloch’s result that TDM represents only the 1% of the surface-extracted lipids, other authors have reported that TDM is not on the surface of *M. tuberculosis* or on the surface of *M. abscessus* (Ortalo-Magné et al., 1996; Burbaud et al., 2016). In accordance with these previous studies, we did not find TDM in PE extracts.

Genetic validation of the link between cording and virulence in *M. tuberculosis* and *M. abscessus* has been obtained through natural and constructed mutants, most of which exhibit alterations in the synthesis or structure of the lipids of the cell wall; thus, they are consistent with the Bloch studies attributing the formation of cords to a lipid substance (Glickman, 2008). Studies performed with genetically defined mutants of *M. tuberculosis* have suggested that cording may be related to the structure of mycolic acids (Glickman et al., 2000). That investigation has attributed the need for a proximal cyclopropane in α-mycolic acids for cording in *M. tuberculosis*. Thus, we found it interesting to investigate the presence of cyclopropane rings in α- and α’-mycolic acids of the non-cording (390S, 44196S) and cording morphotypes (390R, 44196R). Both acids from all sources possessed approximately the same amounts of *cis*-double bonds, and cyclopropane rings were nearly absent. Recently, a cord-deficient *M. abscessus* mutant has been generated, and no differences have been found in the cyclopropanation of mycolic acids between the mutant and the parent strains (Halloum et al., 2016). These results indicate that in *M. abscessus*, the formation of cords is not related to the presence of cyclopropane rings in α and α’-mycolic acids. However, the abovementioned cord-deficient *M. abscessus* mutant exhibits an alteration in its ratio of mycolic acids. The authors have reported that the α-mycolic acid is the major mycolic acid in the parental cording R strain, whereas in the non-cording mutant, the major mycolic acid is the α’-mycolic acid (Halloum et al., 2016). Our results showed no differences in the ratio of mycolic acids in cording and non-cording morphotypes, thus indicating that both morphotypes have a similar amount of α- and α’-mycolic acids. The difference between the results obtained by Halloum et al. (2016) and our results is that they used a mutant deficient in a gene related to mycolic acid biosynthesis, and we used natural morphotypes, that do not seem to have the biosynthesis of mycolic acids affected as measured by TLC and NMR analysis in our assays. Thus, we did not find a correlation between mycolic acids and cording, in accordance with later models of the mycobacterial cell wall that have proposed that these lipids are deeply embedded in the cell wall (Daffé et al., 2014; Jankute et al., 2015). Mycolic acids are major components of the cell envelope of mycobacteria and play a crucial role in its architecture, so we hypothesized that mutants with alterations in mycolic acids might have a modification in their cell wall that causes a change in the cording morphotype, even though mycolic acids do not have a direct role in cord formation.

In the present work, only one major compound, identified as TPP A, was extracted from the surface of R morphotypes but not from the surface of S morphotypes of *M. abscessus* strains. TPP were first characterized in *Mycobacterium phlei* as a trehalose that was acylated with long polyunsaturated fatty acids (called phleic acids because they were also first described in *M. phlei*) (Asselineau et al., 1969, 1972; Asselineau and Montrozier, 1976). Recently, TPP have been detected in *M. smegmatis*, *M. abscessus*, *M. avium*, and other NTM, and those from *M. smegmatis* have been structurally characterized (Burbaud et al., 2016). In this work, we structurally characterized TPP A from the *M. abscessus* 390R strain. We found a major TPP A that was similar to TPP A in *M. smegmatis* and two other minor TPP species (Burbaud et al., 2016).

OsO_4_, a commonly used stain for lipids in electron microscopy, makes the lipids appear as an electrodense zone (Daffé et al., 1989; Bleck et al., 2010). In the images obtained by TEM, a very electrodense material was observed in the outermost layer of the R bacilli and cords. The OTO method particularly enhances the contrast of the lipid components of the cell that were stained by OsO_4_, and with this method, we corroborated the presence of lipids in the external surface of R but not S bacilli (Seligman et al., 1966; Daffé et al., 1989). The obvious candidates for such reactions are TPP, because they were the major lipids extracted from the mycobacterial surface with PE. Consequently, TEM images reinforced the hypothesis that TPP are exposed on the surface of R morphotypes of *M. abscessus* but not on the surface of S morphotypes. Considering this difference, we used beads that were similar in size to bacteria and coated them with TPP A and found that the TPP A-coated beads aggregated more than uncoated beads. When the beads had this compound on their surface, as occurred in R morphotypes of *M. abscessus*, they tended to produce more clumps. TPP are one of the largest known lipids in mycobacteria (Seeliger and Moody, 2016), and their role in the mycobacterial envelope is unknown, but biochemical analyses and microscopy results obtained in this work suggest that TPP may be necessary for the formation of clumps and cords. The very hydrophobic nature of clumps and cords is in accord with the surface exposure of these giant lipids.

We found that TPP are not toxic, by themselves, to the macrophages, do not cause the release of proinflammatory cytokines and do not prevent the phagolysosome fusion. This result is consistent with those of Bloch, who had found that only 1% of the PE extract of cording *M. tuberculosis* is toxic (Bloch et al., 1953). What, then, could be the role of TPP in the increased virulence of *M. abscessus* cording strains? It is reasonable to assume that macrophages that engulf a clump of five or more bacilli in a single phagocytic vesicle should encounter more virulence factors (Brambilla et al., 2016). At present, the majority of these factors are unknown, although the more virulence of R morphotypes has been associated with hyper-proinflammatory responses produced by mycobacterial Toll like receptors ligands as phosphatidyl-myo-inositol mannosides, lipomannan, lipoarabinomannan and lipoproteins (Gilleron et al., 2008; Rhoades et al., 2009; Roux et al., 2011). Therefore, TPP make bacilli aggregate, forming clumps that, when phagocytozed, overwhelm the bactericidal capabilitiesof macrophages. The release of large clumps by damaged macrophages is followed by the formation of cords, a process that protects mycobacteria from phagocytosis (Bernut et al., 2014). Making the cord formation an important determinant of virulence.

In summary, the novel findings reported in this work are: (i) the description for the first time of the fine structure of mycolic acids of cording and non-cording morphotypes derived from the same parent strain of *M. abscessus*; (ii) the confirmation that in natural mutants of *M. abscessus* no differences exist in mycolic acid composition between cording and non-cording morphotypes; (iii) the structural characterization of TPP in *M. abscessus* strains; (iv) the location of lipidic compounds on the surface of cording R morphotypes of *M. abscessus* but not in the surface of non-cording S morphotypes; (v) the determination of the aggregative capacity of TPP; and (vi) the determination of the no toxicity of TPP for macrophages. All these findings allow us to propose TPP as candidate molecules responsible for the formation of clumps and cords in R *M. abscessus* strains.

Because the *papA3*, *pks*, *fadD23*, and *mmpL10* genes have recently been found to be involved in the biosynthesis and transport of TPP (Burbaud et al., 2016), it will be possible to confirm the role of TPP in cording-defective mutants. With regard to *M. tuberculosis*, no TPP have been described in this species, but other acylated trehaloses that are closely related in structure to TPP (sulfolipids, diacyltrehaloses, and polyacyltrehaloses) are present in the cell walls of this species and related with hydrophobicity and pathogenicity (Daffé et al., 2014; Jankute et al., 2015, 2017). Future studies are necessary to clarify the role of these acylated trehaloses in the cording of *M. tuberculosis*.

## Author Contributions

Conceived and designed the experiments: ML-F, EJ, and ML. Performed the experiments: ML-F, MP-T, and CB. Analyzed the data ML-F, MP-T, EJ, and ML. Contributed reagents/materials/analysis tools TB and FA. Contributed to the writing of the manuscript: ML-F, MP-T, EJ, CB, TB, FA, and ML.

## Conflict of Interest Statement

The authors declare that the research was conducted in the absence of any commercial or financial relationships that could be construed as a potential conflict of interest.

## Abbreviations

BSA: bovine serum albumin
CLSM: confocal laser scanning microscope
CM: complete medium
DMEM: Dulbecco’s Modified Eagle’s Medium
DMSO: dimethyl sulfoxide
FA: formaldehyde
GA: glutaraldehyde
MS: mass spectrometry
MTT: 3-(4,5-dimethylthiazol-2-yl)-2,5-diphenyltetrazolium bromide
NMR: nuclear magnetic resonance
NTM: nontuberculous mycobacteria
OsO_4_: osmium tetroxide
PBS: phosphate-buffered saline
PE: petroleum ether
RPMI: Roswell Park Memorial Institute medium
SEM: scanning electron microscope
TAG: triacylglycerides
TDM: trehalose dimycolate
TEM: transmission electron microscope
TLC: thin layer chromatography
TPP: trehalose polyphleates
