# Supplementary material for: Trehalose Polyphleates, External Cell Wall Lipids in Mycobacterium abscessus, Are Associated with the Formation of Clumps with Cording Morphology, Which Have Been Associated with Virulence
Source: Front Microbiol. 2017 Jul 25;8:1402. doi: 10.3389/fmicb.2017.01402 (PMC5524727; doi:10.3389/fmicb.2017.01402)
Supplement: Supplementary file 1 [file Data_Sheet_1.PDF]

*Supplementary Material*

# Trehalose polyphleates, external cell wall lipids in *Mycobacterium abscessus*, are associated with the formation of clumps with cording morphology, which have been associated with virulence

**Marta Llorens-Fons, Míriam Pérez-Trujillo, Esther Julián, Cecilia Brambilla, Fernando Alcaide, Thomas F. Byrd, Marina Luquin***

***Corresponding author**: Marina Luquin (mailto: marina.luquin@uab.cat)

## 1 Supplementary Figures and Tables

### 1.1 Supplementary Figures

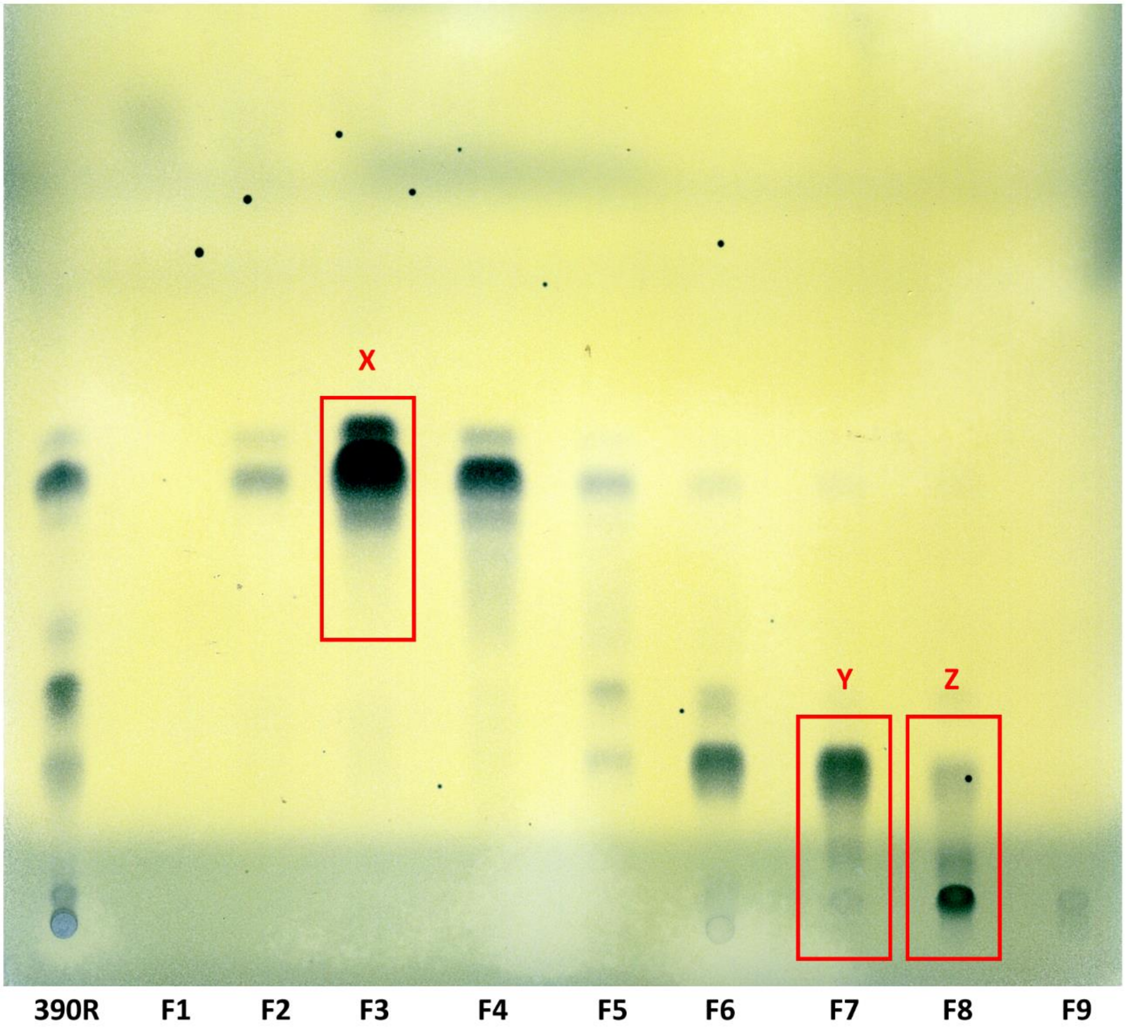


**Figure S1.** TLC of all the fractions (F1-F9) obtained when performing a column chromatography to the PE extract from 390R *M. abscessus*. The solvent system used was PE 60-80°C/diethyl ether (90:10, v/v), and the plate was stained with 10% phosphomolybdic acid. Fractions 3, 7 and 8, corresponding to compound X, compound Y and compound Z, were analyzed by NMR and MS.

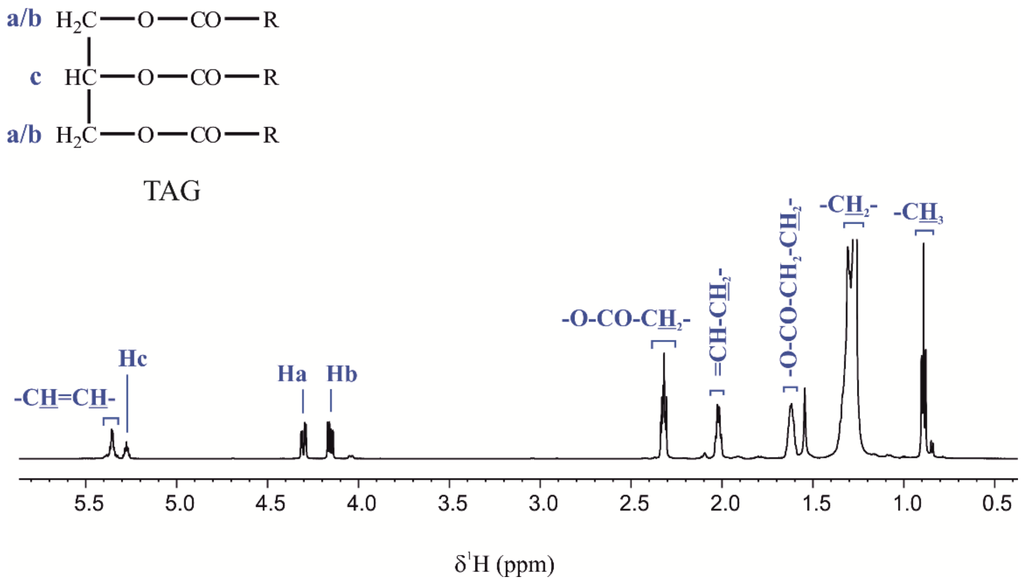


**Figure S2.** $^1$H NMR spectrum of TAG from PE extract of 390R *M. abscessus* strain in $CDCl_3$, at a magnetic field of 600.13 MHz and 298.0 K of temperature.

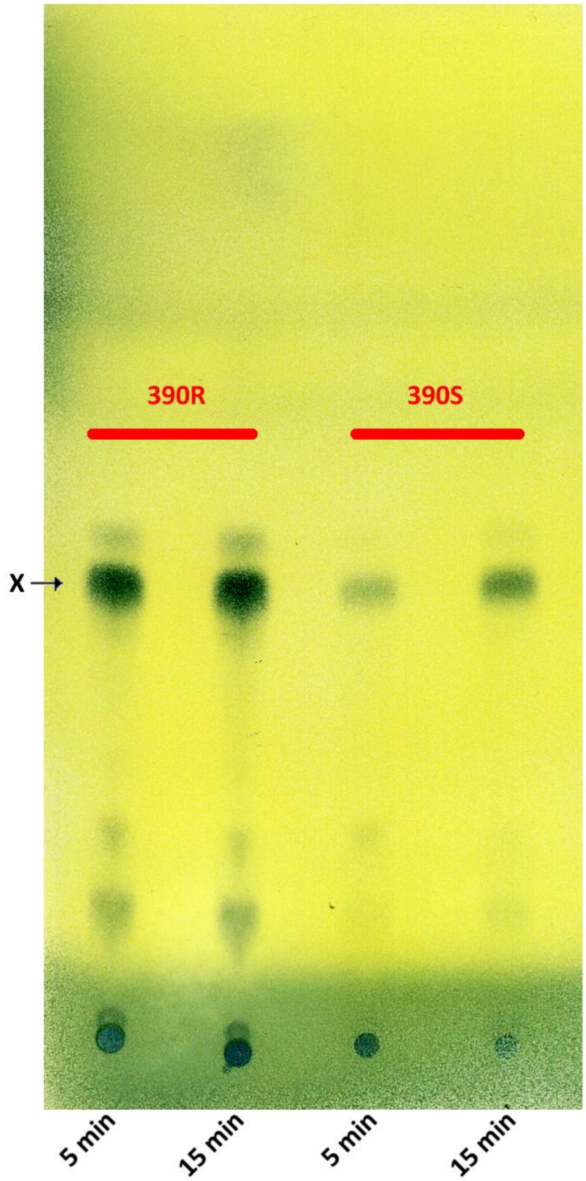


**Figure S3.** TLC of the PE extracts obtained from 390R and 390S after 5 minutes and after 15 minutes of extraction. The solvent system used was PE 60-80°C/diethyl ether (90:10, v/v), and the plate was revealed with 10% phosphomolybdic acid. X indicate compound X or TPP-A.

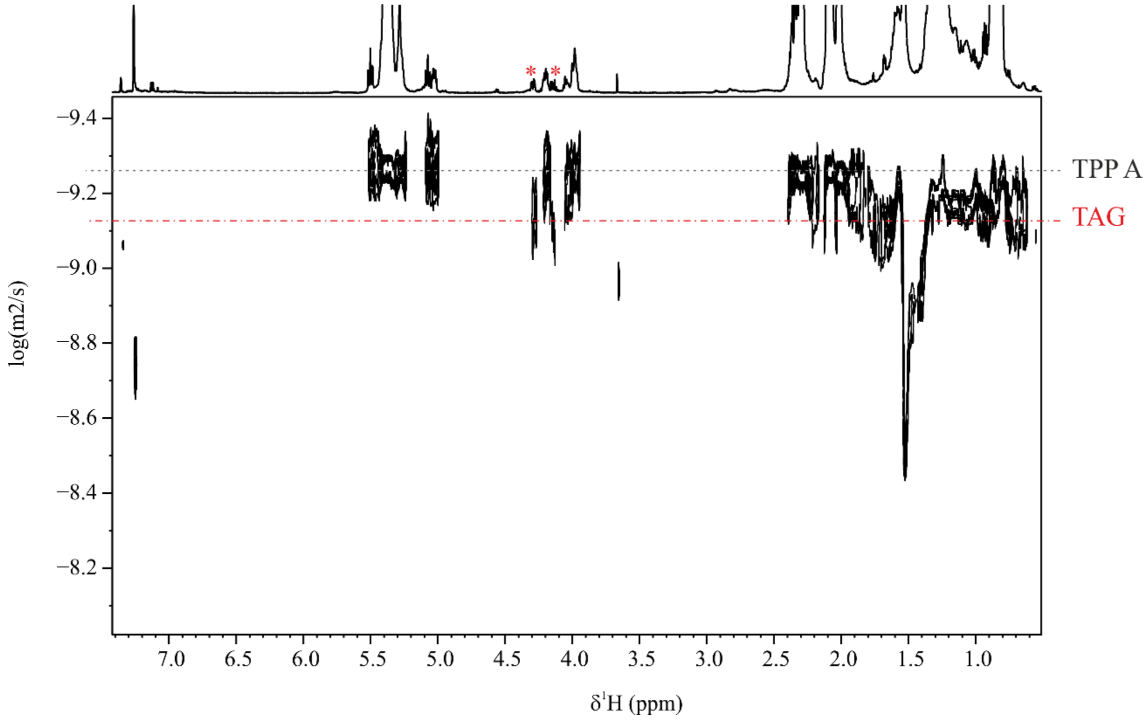


**Figure S4.** 2D DOSY spectrum of TPP-A from PE extract of 390R *M. abscessus* strain in $CDCl_3$ at a magnetic field of 600.13 MHz and 298.0 K of temperature; asterisks denote signals of TAG.

**A**

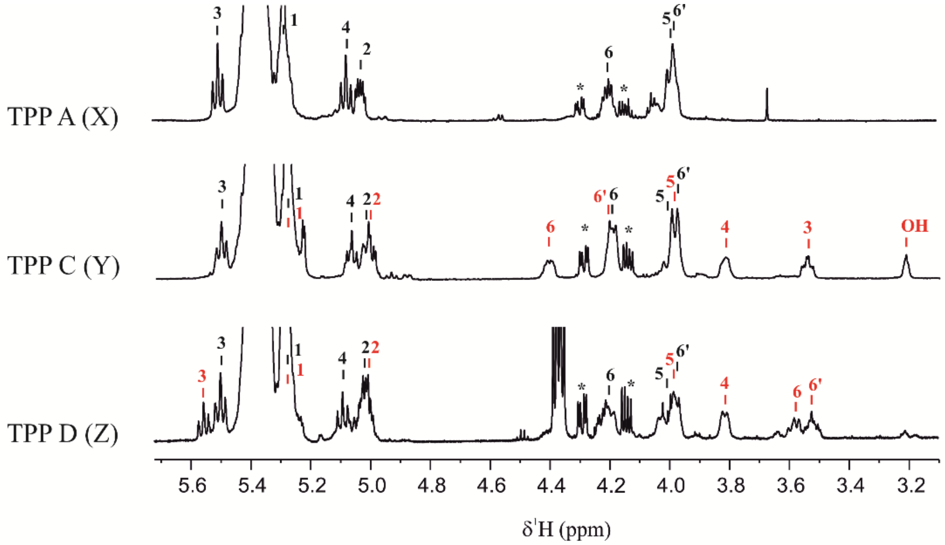


**B**

| Trehalose | Id | TPP A (X) δ¹H | TPP A (X) δ¹³C | TPP C (Y) δ¹H | TPP C (Y) δ¹³C | TPP D (Z) δ¹H | TPP D (Z) δ¹³C |
|---|---|---|---|---|---|---|---|
| Glucosyl 1 | 1 | 5.28 (d) | 91.6 | Idem TPP A | | Idem TPP A | |
| | 2 | 5.03 (dd) | 69.7 | | | | |
| | 3 | 5.50 (t) | 70.0 | | | | |
| | 4 | 5.07 (t) | 68.0 | | | | |
| | 5 | 3.97 (m) | 68.1 | | | | |
| | 6 | 4.20 | 61.3 | | | | |
| | 6' | 3.98 | 61.3 | | | | |
| Glucosyl 2 | 1 | Idem Glucosyl 1 | | 5.28 (d) | 91.6 | 5.28 (d) | 92.1 |
| | 2 | | | 5.01 (m) | 69.7 | 5.02 (m) | 69.8 |
| | 3 | | | 3.54 (t) | 69.4 | 5.56 (t) | 69.4 |
| | 4 | | | 3.82 (m) | 70.7 | 3.82 (m) | 70.5 |
| | 5 | | | 3.99 (m) | 68.1 | 4.01 (m) | 68.1 |
| | 6 | | | 4.40 | 62.3 | 3.58 | 61.0 |
| | 6' | | | 4.19 | 62.3 | 3.53 | 61.0 |

**C**

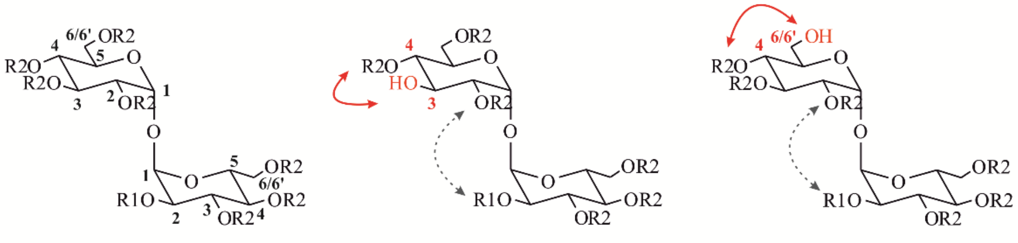


TPP A (X) TPP C (Y) TPP D (Z)

**Figure S5. (A)** $^{1}$H NMR spectra in $CDCl_3$ (enlargement of the region between 5.7 and 3.4 ppm); asterisks denote signals of TAG; **(B)** $^{1}$H and $^{13}$C NMR characterization of the trehalose units and **(C)** suggested structures for TPP A (compound X), TPP C (compound Y) and TPP D (compound Z).

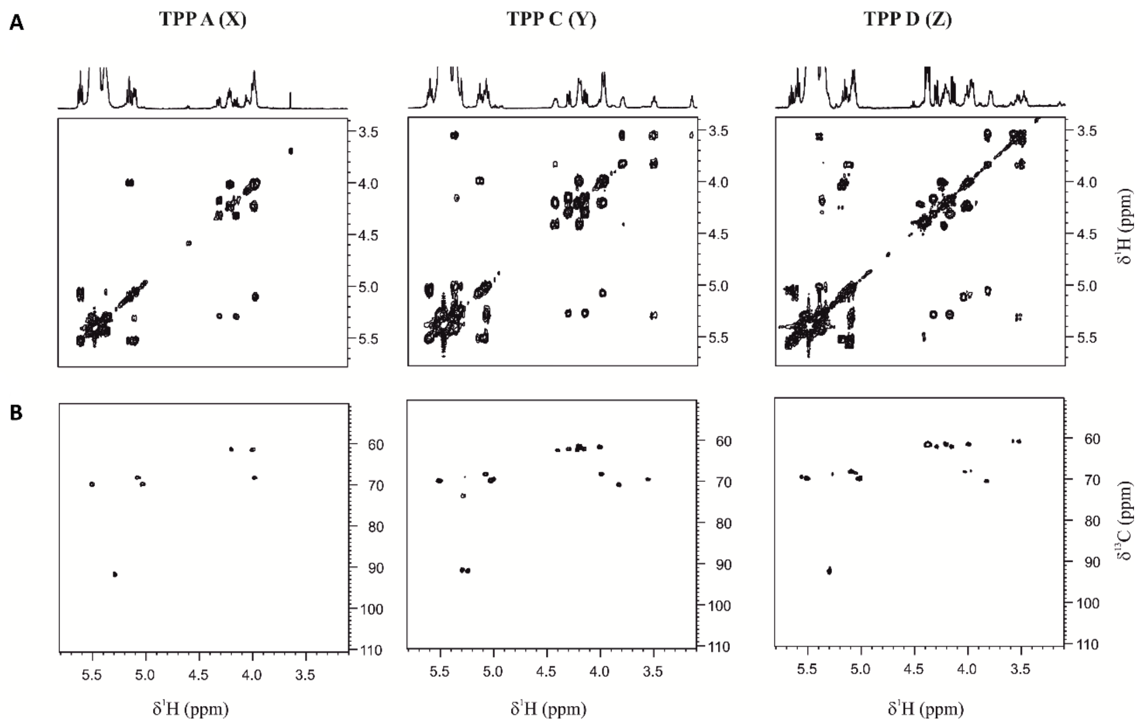


**Figure S6. (A)** 2D $^{1}H$,$^{1}H$-COSY and **(B)** $^{1}H$,$^{13}C$-HSQC of TPP A (compound X), TPP C (compound Y) and TPP D (compound Z) in $CDCl_3$

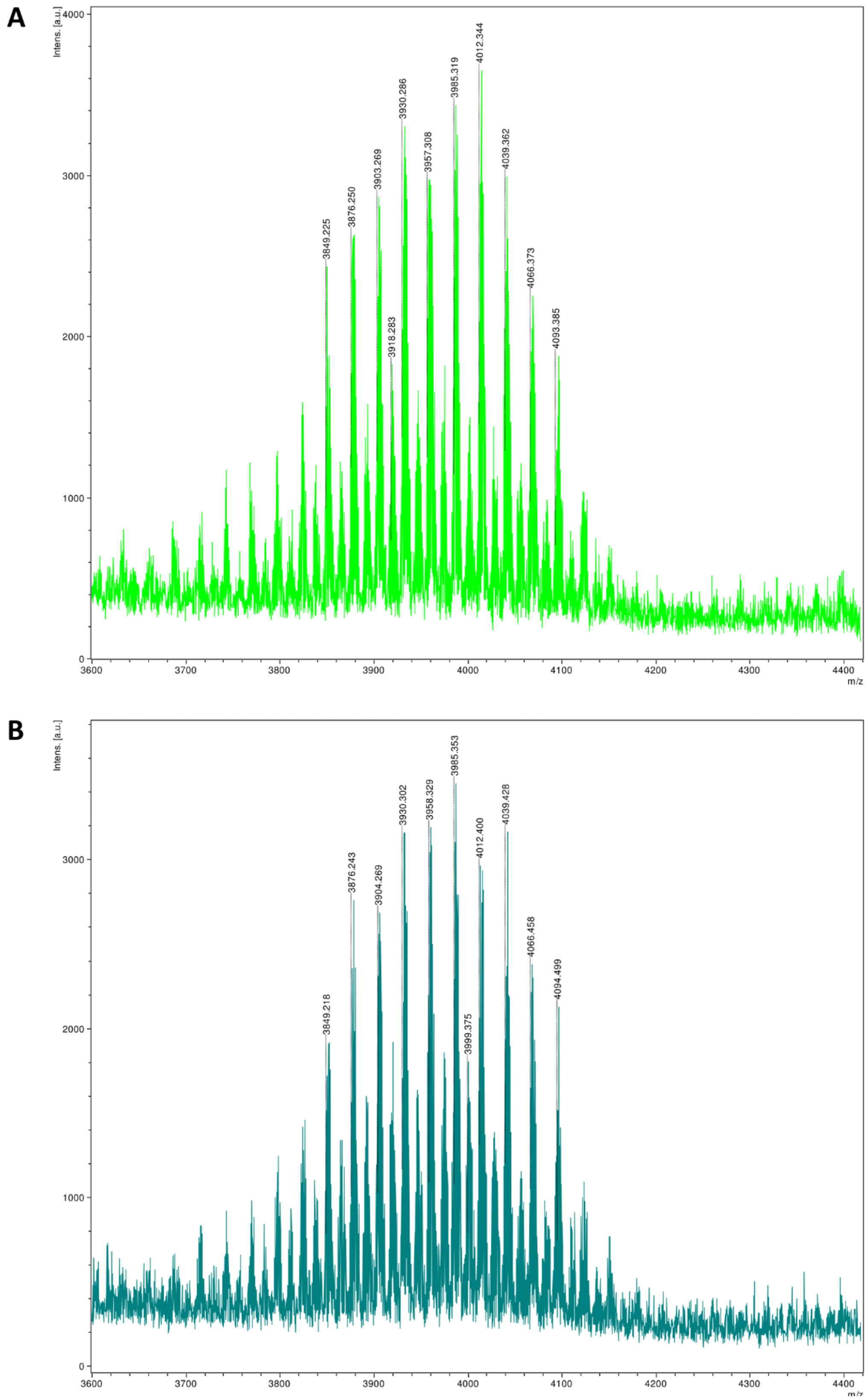


**Figure S7.** MALDI-TOF MS spectra of **(A)** TPP C (compound Y) and **(B)** TPP D (compound Z) (region between m/z 3600 and 4400 was magnified).
